# Substitution of the myristoylation signal of human immunodeficiency virus type 1 Pr55^Gag^ with the phospholipase C-*δ*1 pleckstrin homology domain results in infectious pseudovirion production

**DOI:** 10.1099/vir.0.2008/004820-0

**Published:** 2008-12

**Authors:** Emiko Urano, Toru Aoki, Yuko Futahashi, Tsutomu Murakami, Yuko Morikawa, Naoki Yamamoto, Jun Komano

**Affiliations:** 1 AIDS Research Center, National Institute of Infectious Diseases, 1-23-1 Toyama, Shinjuku-ku, Tokyo 162-8640, Japan; 2 Kitasato Institute of Life Sciences, Kitasato University, Shirokane 5-9-1, Minato-ku, Tokyo 108-8641, Japan

## Abstract

The matrix domain (MA) of human immunodeficiency virus type 1 Pr55^Gag^ is covalently modified with a myristoyl group that mediates efficient viral production. However, the role of myristoylation, particularly in the viral entry process, remains uninvestigated. This study replaced the myristoylation signal of MA with a well-studied phosphatidylinositol 4,5-biphosphate-binding plasma membrane (PM) targeting motif, the phospholipase C-*δ*1 pleckstrin homology (PH) domain. PH–Gag–Pol PM targeting and viral production efficiencies were improved compared with Gag–Pol, consistent with the estimated increases in Gag–PM affinity. Both virions were recovered in similar sucrose density-gradient fractions and had similar mature virion morphologies. Importantly, PH–Gag–Pol and Gag–Pol pseudovirions had almost identical infectivity, suggesting a dispensable role for myristoylation in the virus life cycle. PH–Gag–Pol might be useful in separating the myristoylation-dependent processes from the myristoylation-independent processes. This the first report demonstrating infectious pseudovirion production without myristoylated Pr55^Gag^.

The N-terminal region [p17^MA^, matrix (MA) domain] of human immunodeficiency virus type 1 (HIV-1) Pr55^Gag^ (Gag), a structural protein with multiple roles in the virus life cycle (Swanstrom & Wills, 1997), is covalently modified with a myristyol group that aids in plasma membrane (PM) targeting. Removal of this region leads to inefficient Gag targeting to the PM, resulting in dramatically reduced virus production (Bryant & Ratner, 1990; Göttlinger *et al.*, 1989; Pal *et al.*, 1990; Zhou *et al.*, 1994). Although viral particles can be produced by substituting MA with heterologous PM-targeting motifs, such substitution mutants show markedly reduced infectivity (Jouvenet *et al.*, 2006; Scholz *et al.*, 2008), probably due to an active role of MA in viral entry (Kiernan *et al.*, 1998; Wang *et al.*, 1993). However, direct experimental evidence of a viral entry-specific role for MA myristoylation is lacking. Such specific roles of Gag myristoylation can only be determined by separating the myristoylation-dependent PM-targeting function from other MA-associated functions.

We constructed a mutant *gag* expression plasmid where the myristoylated region of Gag was replaced with the N-terminal pleckstrin homology (PH) domain of phospholipase C-*δ*1 (PLC*δ*1), a well-studied cellular PM-targeting motif that functions similarly to the myristoyl moiety. PLC*δ*1 is a member of a family of inositol phospholipid-specific PLC isozymes involved in transducer-mediated intracellular responses (Berridge, 1993). The ∼120 aa PH domain can bind to phosphatidylinositol 4,5-biphosphate [PI(4,5)P(2)] and localize to the PM with high affinity and specificity (Ferguson *et al.*, 1995b; Fiorentini *et al.*, 2006; Harlan *et al.*, 1994, 1995; Rhee, 2001; Yagisawa *et al.*, 1994), and green fluorescent protein-bound PLC*δ*1 PH domains have been used to visualize the PM in living cells (Stauffer *et al.*, 1998; Tall *et al.*, 2000).

We used a codon-optimized HIV-1 *gag–pol* expression vector (p*gag–pol*) for genetic modification of *gag*, as p*gag–pol* increases Gag expression and facilitates protein analyses (Wagner *et al.*, 2000). The substituted mutant retained an intact MA, with the exception of two N-terminal amino acid mutations (ATG→CTG and GGC→GCG), resulting in an MG→LA mutation to knock out the myristoylation signal of Gag and prevent internal translational initiation (Fig. 1a). The PLC*δ*1 PH domain residues 1–175 (Stauffer *et al.*, 1998) were linked to the LA–Gag N terminus by the amino acids PRAEFT, creating a PH–*gag–pol* expression vector (pPH-*gag-pol*, Fig. 1a). A control PH domain mutant (PH4A) had mutations at aa 54–57 (ESRK→AAAA; Fig. 1a); these residues are responsible for the PH domain–PI(4,5)P(2) interaction (Ferguson *et al.*, 1995a). PH–Gag, PH4A–Gag and their cleaved products were detected in transfected 293T cell lysates with mouse monoclonal antibodies specific for the p24^CA^ (capsid) domain (anti-p24^CA^; NIH AIDS Research and Reference Reagent Program) and MA domain (anti-p17^MA^; Advanced Biotechnologies) (Fig. 1b). PH–Gag cleavage was more efficient than that of Gag, suggesting efficient PM targeting of PH–Gag (Fig. 1b). The Gag protein levels in the pPH4A-*gag-pol*-transfected cell lysate were higher than those in p*gag-pol*- and pPH-*gag-pol*-transfected cell lysates when adjusted for the amount of protein loaded, indicating the low virus-like particle (VLP) production efficiency by PH4A–Gag (Fig. 1b).

The intracellular distribution of Gag, PH–Gag and PH4A–Gag was analysed by immunofluorescence microscopy of transfected 293T cells (Fig. 1c). Transfected cells were grown for 24 h, fixed (4 % formaldehyde), permeabilized (0.1 % Triton X-100 for 5–30 min) and incubated with mouse anti-p24^CA^ and goat anti-mouse antibodies (GE Healthcare Bio-Sciences) conjugated to streptavidin–Alexa Fluor 555 (Invitrogen). Cells were stained with Hoechst 33258, mounted and analysed using confocal microscopy as described previously (Futahashi *et al.*, 2007). Gag was found to be distributed throughout the cytoplasm and at the cell periphery. In contrast, PH–Gag signals were mostly detected at the cell periphery and PH4A–Gag was distributed homogeneously in the cytoplasm. These data clearly showed that PH–Gag targeted the PM more efficiently than Gag, consistent with the Western blot analysis (Fig. 1b). These results also suggested that the efficient PM targeting of PH–Gag depends on the ability of the PH domain to bind PI(4,5)P(2). Similar observations were made in NP2 and COS7 cells.

VLP production was also examined. Tissue culture supernatants of p*gag-pol*-, pPH-*gag-pol*- or pPH4A-*gag-pol*-transfected 293T cells were passed through nitrocellulose filters (0.45 μm) and the virions were collected by centrifugation (541 000 ***g*** for 1 h). Viral antigens, except for PH4A, were detected with anti-p24^CA^ and anti-p17^MA^ antibodies (Fig. 1b). Gag and PH–Gag were further processed by the viral proteases in the virions compared with the cell lysates, as indicated by the increased signals for CA and MA relative to Gag. Interestingly, approximately one-fifth of the PH–Gag in the virion was cleaved close to the PH–MA junction. Presumably, the amino acid sequence at the C end of the PH domain ELQN/FLKE (aa 164–171, where the protease cleaves at the N–F junction) served as a viral protease recognition site as it matched the substrate consensus sequence and resembled the NC–*p1* junction, RQAN/FLGK (de Oliveira *et al.*, 2003; Swanstrom & Wills, 1997). Alternatively, the N terminus of LA–Gag (EFTL/AADS) might be targeted by the viral protease. Thus, the MA released from PH–MA, designated MA*, possibly has 10 aa attached to its N terminus.

The VLP production efficiency was quantified as the concentration of CA in transfected 293T cell culture supernatants relative to that in cell lysates using a p24 ELISA (Zeptometrics). When the CA concentrations of the virion fractions were normalized to those of the cell lysates, the pPH-*gag-pol* viral production efficiency was 3.2-fold higher than that of p*gag-pol* (3.2±2.0-fold, *n*=14, *P*<0.001 by Wilcoxon's matched pairs rank test; representative experiments are shown in Table 1). In contrast, pPH4A-*gag-pol* produced viral particles less efficiently than p*gag-pol* (0.09±0.07-fold, *n*=6, *P*<0.05 by Wilcoxon's matched pairs rank test; representative experiments are shown in Table 1). These data were consistent with the Western blot analysis (Fig. 1b).

To characterize the physical properties of PH–Gag–Pol VLPs, we measured the specific density of virions and examined the virion morphology. Firstly, the VLPs were subjected to 20–70 % (w/w) equilibrium sucrose gradient centrifugation (120 000 ***g*** for 16 h) and fractions were recovered from the bottom to the top. The peak fraction containing the viral CA antigen was determined by p24 ELISA. Gag–Pol and PH–Gag–Pol VLPs were detected in fractions with densities of 1.15±0.01 (*n*=5) and 1.16±0.01 (*n*=4) g ml^−1^, respectively (not statistically significant; representative experiments are shown in Fig. 2a). Secondly, ultrathin sections of fixed 293T cells (2 % glutaraldehyde, 2 % osmium tetroxide) transfected with p*gag-pol* or pPH-*gag-pol* were imaged by transmission electron microscopy (JEM1200EX at 80 kV or JEM2000EX at 100 kV; JEOL). The PH–Gag–Pol and Gag–Pol VLP diameters were almost identical (Fig. 2b). Virion budding structures showed that the electron-dense layer, which represented multimerized Gag, of the PH–Gag–Pol VLP was slightly separated from the viral envelope compared with that of the Gag–Pol VLP (Fig. 2b). This indicated that the PH domain was positioned between the viral envelope and the electron-dense layer. In contrast, the morphologies of the mature PH–Gag–Pol and Gag–Pol virions were similar, suggesting that myristoylation is dispensable for mature virion morphology and that the PH–Gag–Pol virion may be infectious.

We examined HIV-1 Env incorporation into the PH–Gag–Pol virion. To do this, we used codon-optimized gp160 (p96ZM651gp160-opt; NIH AIDS Research and Reference Reagent Program). The PH–Gag–Pol virion incorporated HIV-1 Env less efficiently than the Gag–Pol virion as demonstrated by Western blot analysis detecting CA and gp120 (anti-gp120 antibodies from Santa Cruz Biotechnology; Fig. 2c). This was presumably because PH interfered with the MA–Env interaction. Alternatively, PH may actively incorporate cellular proteins that block efficient Env incorporation into virions. We were unable to evaluate the entry efficacy of PH–Gag–Pol virions pseudotyped by HIV-1 Env because of the limit of detection. PH–Gag–Pol virion infectivity was re-examined by virions pseudotyped with vesicular stomatitis virus G glycoprotein (VSV-G). The incorporation efficiencies of VSV-G into Gag–Pol and PH–Gag–Pol virions were similar (anti-VSV-G antibody from Sigma; Fig. 2c). The lentiviral vector system was used to test this, as HIV-1 provirus gene modifications often fail to produce infectious virions, probably due to viral gene dysregulation. 293T cells were transfected with expression plasmids for Gag–Pol, VSV-G (Komano *et al.*, 2004), Rev and Vpu (a generous gift from Dr H. Göttlinger, University of Massachusetts Medical School, MA, USA), and with a packaging vector encoding a luciferase expression cassette. The HIV-1-based vector expressing firefly luciferase upon infection was recovered 2 days post-transfection. 293T cells were exposed to virus-containing culture supernatants with similar CA concentrations, and luciferase activities were measured at 2–3 days post-infection. When viral preparations with similar p24 concentrations were used, the luciferase activities of PH–Gag–Pol and Gag–Pol virus-infected cells were almost identical to each other (Fig. 2d, left graph). Luciferase expression was blocked by the non-nucleoside reverse transcriptase inhibitor nevirapine (NVP; Boehringer Ingelheim) but not by the CCR5 inhibitor (TAK-779; NIH AIDS Research and Reference Reagent Program), suggesting that gene transduction was mediated by viral infection (Fig. 2d, right panel). Similar results were obtained in several independent experiments. Thus, the PLC*δ*1 PH domain can functionally replace the HIV-1 Gag myristoylation signal to support both viral production and entry processes, and this myristoylation is dispensable for MA function in the early phase of the virus life cycle. This is the first report describing an infectious pseudovirion without myristoylated Gag. Given that PH–Gag can enhance virus production, HIV-1 with PH–Gag might have been expected to be selected in nature. This is not the case, presumably because the addition of PH to the HIV-1 genome would increase its genome size close to the upper limit that can be incorporated into the retroviral particle, leading to a decrease in genome uptake efficiency, which is clearly a growth disadvantage, despite the enhanced virus production with PH-Gag. More importantly, PH–Gag is unable to incorporate HIV-1 Env efficiently enough to support the production of fully infectious virions. Our data point to the selective advantage of myristoylated Gag in viral evolution.

The myristoylation-dependent Gag–PM association [maximal dissociation constant (*K*_d_) of ∼0.5–1.0×10^−5^ M] is presumably important for Gag multimerization at the PM (Provitera *et al.*, 2006). After the first contact of Gag with the PM, the membrane binding of Gag is assumed to be stabilized by the Gag–PI(4,5)P(2) interaction (Ono *et al.*, 2004; Saad *et al.*, 2006). The multimerization of Gag appears to induce a conformational change in MA to expose myristoyl groups to enhance the PM targeting of Gag. The higher-order Gag multimerization is probably facilitated by the increased local concentrations of Gag at the PM. Although Gag and PH–Gag are similar to the extent that PI(4,5)P(2) is involved in their PM association, Gag binds to one of the acyl chains of PI(4,5)P(2), as modelled previously (Saad *et al.*, 2006), whilst the PH domain binds the phosphorylated inositol group (Lemmon *et al.*, 1995). The *K*_d_ of binding between the PLC*δ*1 PH domain and PI(4,5)P(2) (∼1–2×10^−6^ M; Lemmon *et al.*, 1995) suggests that the primary force driving PH–Gag to the PM is at least 2.5-fold stronger than that of myristoylation-mediated PM targeting of Gag. This might be one reason why PH–Gag–Pol was 3.2-fold more efficient at virion production than Gag–Pol. Our data suggest that the myristoyl group-dependent Gag–PM affinity is not a prerequisite for efficient Gag assembly at the PM or for viral production.

The MA has multiple functions throughout the virus life cycle (reviewed by Bukrinskaya, 2007; Fiorentini *et al.*, 2006; Hearps & Jans, 2007; Klein *et al.*, 2007). In the PH–Gag–Pol virion, approximately one-fifth of the PH–MA was unanchored from the PM as MA* (Fig. 1b), which might accompany the pre-integration complex to support nuclear targeting. Using PH–Gag–Pol might enable separation of myristoylation-dependent and -independent MA functions, particularly during the entry phase. PH–Gag–Pol might also be useful for producing high-titre lentiviral vectors or for studying Gag trafficking in cells that poorly support PM targeting of myristoylated Gag, such as rodent cells. Furthermore, functional assays comparing the virus production of Gag–Pol and PH–Gag–Pol might enable the identification of chemical inhibitors or cellular factors specifically targeting myristoylated Gag.

## Figures and Tables

**Fig. 1.**
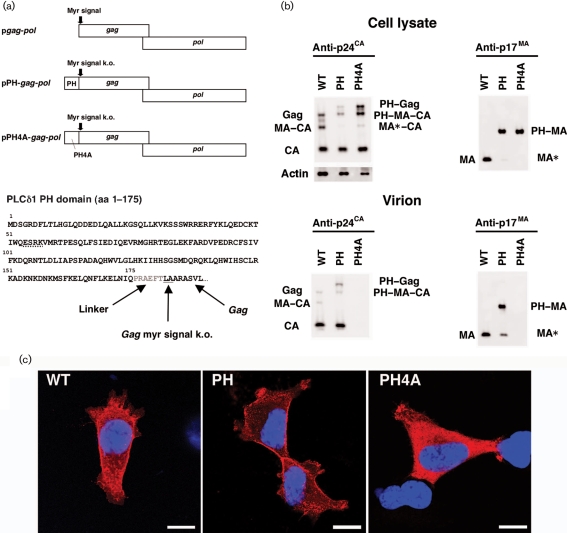
Viral production by the *gag–pol* expression vectors. (a) The genetic structure of the p*gag-pol*, pPH-*gag-pol* and pPH4A-*gag-pol* expression vectors, and the amino acid sequence of the PH–Gag junction are shown. The N-terminal PH domain of PLC*δ*1 (aa 1–175) was fused to LA–Gag, linked by a 5 aa spacer (shown in grey). The MG→LA mutation to knock out the myristoylation signal of Gag (myr signal k.o.) is underlined. Four alanine mutations were introduced to replace the ESRK sequence (dotted line) to create the PH4A mutant. (b) Protein expression from p*gag-pol* (WT), pPH-*gag-pol* (PH) and pPH4A-*gag-pol* (PH4A) in transfected 293T cell lysates and Gag cleavage in the virions were examined by Western blot analysis using anti-p24^CA^ or anti-p17^MA^ antibodies. Note that the anti-p17^MA^ antibody recognizes the cleaved p17^MA^ protein only. The band denoted as PH–MA–CA in the virion detected by the anti-p24^CA^ antibody (lower left panel) possibly overlaps with a faint Gag signal derived from PH–Gag and PH4A–Gag from which the PH and PH4A domains have been cleaved. (c) Immunofluorescence assay showing the distribution of Gag, PH–Gag and PH4A–Gag in 293T cells transfected with the respective expression plasmid. Red and blue represent p24^CA^ and the Hoechst 33258-stained nucleus, respectively. Bars, 10 μm.

**Fig. 2.**
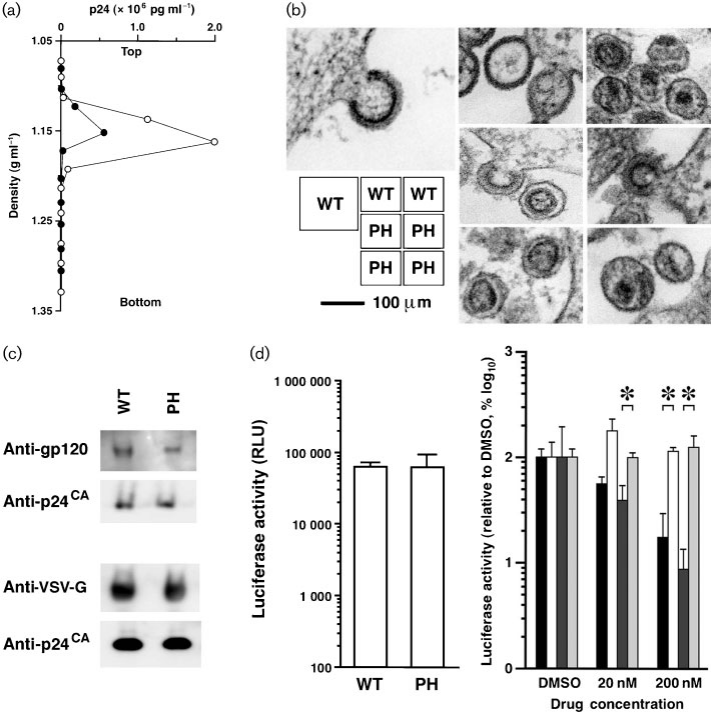
Physical and biological properties of PH–Gag–Pol VLPs. (a) The virions produced by the p*gag-pol* (WT) and pPH-*gag-pol* (PH) expression vectors were analysed by equilibrium sucrose density-gradient centrifugation. The virion-containing fraction was determined by an ELISA detecting p24^CA^. Representative data from four to five independent experiments are shown. In this experiment, WT (filled circles) and PH (open circles) VLPs migrated in the 1.152 or 1.162 g ml^−1^ density fractions, respectively. (b) Transmission electron microscopy images of 293T cells transfected with p*gag-pol* (WT) or pPH-*gag-pol* (PH). Representative images are shown. (c) Incorporation of HIV-1 Env (upper panel) and VSV-G (lower panel) into Gag–Pol (WT) and PH–Gag–Pol (PH) virions. The virion fractions were subjected to Western blot analysis detecting gp120, VSV-G and p24^CA^. (d) The early phase of the HIV-1 life cycle is supported by PH–Gag–Pol. 293T cells were exposed to virus-containing culture supernatants with similar CA concentrations (270 and 220 ng ml^−1^ for Gag–Pol and PH–Gag–Pol, respectively), and luciferase activities were measured at 2–3 days post-infection as relative light units (RLU). The luciferase activities of PH–Gag–Pol (PH) and Gag–Pol (WT) virus-infected cells were almost identical (left graph). The luciferase transduction by WT (bars 1 and 2) and PH (bars 3 and 4) pseudovirions was performed in the presence of nevirapine (NVP, bars 1 and 3) or TAK-779 (bars 2 and 4). The luciferase signals decreased in the presence of NVP for both WT (bar 1) and PH (bar 3) but not in the presence of TAK-779 for both WT (bar 2) and PH (bar 4), respectively. Representative data from several independent experiments are shown. Asterisks indicate statistical significance (*P*<0.01, *n*=3, Student's *t*-test).

**Table 1.** Efficiency of virus production from 293T cells transfected with p*gag-pol*, pPH-*gag-pol* or pPH4A*-gag-pol* expression vector

**Experiment**	**Plasmid**	p24^CA^ (ng per well)*		**Virus production efficiency (B/A)**	**Fold increase relative to p*gag-pol***
		**Cell lysate (A)**	**Culture supernatant (B)**		
1	p*gag-pol*	4 043	4 869	1.204	–
	pPH-*gag-pol*	1 989	8 363	4.206	3.49
	pPH4A-*gag-pol*	3 175	103	0.033	0.03
2	p*gag-pol*	3 521	3 887	1.104	–
	pPH-*gag-pol*	2 638	7 688	2.914	2.64
	pPH4A-*gag-pol*	5 913	1 125	0.190	0.17
3	p*gag-pol*	3 359	4 160	1.239	–
	pPH-*gag-pol*	1 454	5 172	3.558	2.87
	pPH4A-*gag-pol*	4 226	75	0.018	0.01
4	p*gag-pol*	9 666	8 996	0.931	–
	pPH-*gag-pol*	4 699	18 273	3.889	4.18
	pPH4A-*gag-pol*	5 527	534	0.097	0.10

*Cells grown in six-well plates were transfected using Lipofectamine 2000 according to the manufacturer's protocol (Invitrogen).
